# p62/SQSTM1: ‘Jack of all trades’ in health and cancer

**DOI:** 10.1111/febs.14712

**Published:** 2018-12-18

**Authors:** Pablo Sánchez‐Martín, Tetsuya Saito, Masaaki Komatsu

**Affiliations:** ^1^ Department of Biochemistry Niigata University Graduate School of Medical and Dental Sciences Japan; ^2^ Department of Physiology Juntendo University Graduate School of Medicine Tokyo Japan

**Keywords:** autophagy, cancer, Keap1, mTORC1, NF‐κB, Nrf2, p62/SQSTM1

## Abstract

p62 is a stress‐inducible protein able to change among binding partners, cellular localizations and form liquid droplet structures in a context‐dependent manner. This protein is mainly defined as a cargo receptor for selective autophagy, a process that allows the degradation of detrimental and unnecessary components through the lysosome. Besides this role, its ability to interact with multiple binding partners allows p62 to act as a main regulator of the activation of the Nrf2, mTORC1, and NF‐κB signaling pathways, linking p62 to the oxidative defense system, nutrient sensing, and inflammation, respectively. In the present review, we will present the molecular mechanisms behind the control p62 exerts over these pathways, their interconnection and how their deregulation contributes to cancer progression.

## Graphical abstract

p62/SQSTM1 interacts with key regulator proteins for diverse signal transduction pathways including mTORC1 activation, NF‐κB signaling, the Keap1‐Nrf2 system, and selective autophagy and serves as a signaling hub for anabolism, inflammatory response, antioxidant response, and catabolism. In this State‐of‐the‐Art Review, we discuss about diverse roles of p62/SQSTM1 and about their deregulation in cancer.

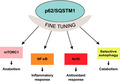

**Abbreviations**
ARE: antioxidant response element
bZIP: leucine zipper
ccRCC: clear cell renal cell carcinoma
CK2: casein kinase 2
CLEAR: coordinated lysosomal expression regulation
CNC: cap‘n'collar
EpRE: electrophile response element
HCC: hepatocellular carcinoma
KIR: Keap1‐interacting region
LIR: LC3‐interacting region
mTORC1: mechanistic target of rapamycin complex 1
PB1: Phox1 and Bem1p
PDAC: pancreatic ductal adenocarcinoma
ROS: reactive oxygen species
TAK1: TGF‐β‐activated kinase
TBK1: TANK‐binding kinase 1
TB: TRAF6 binding
TRAF6: tumor necrosis factor receptor‐associated factor 6
UBA: ubiquitin‐associated
ZZ: zinc finger

## Introduction

**Figure 1:**
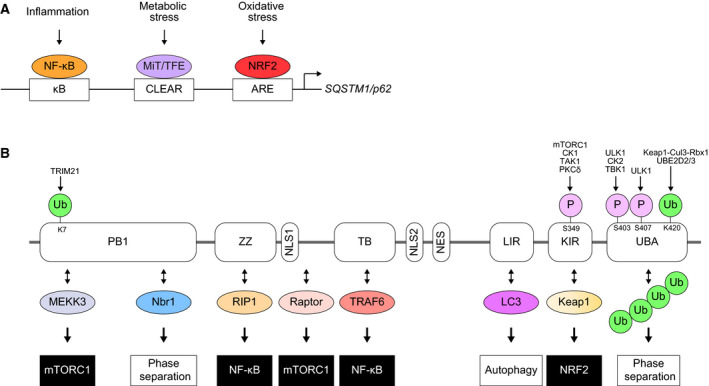
(A) Gene expression of *SQSTM1/p62* through stress‐responsible transcription factors. CLEAR: coordinated lysosomal expression regulation**.** ARE: antioxidant response element. **(**B) Domain structure of the p62 protein. Through the interaction with multiple proteins, p62 serves as a signaling hub that modulates a variety of cellular functions (anabolism and catabolism) and/or protein property (phase separation), which are regulated by post‐translational modifications of p62. PB1: Phox and Bem1p. ZZ: Zinc finger. NLS: nuclear localization signal. TB: TRAF6‐binding domain. NES: nuclear export signal. LIR: LC3‐interacting region. KIR: Keap1‐interacting region. UBA: ubiquitin‐associated.

*SQSTM1*, the human gene encoding for p62/SQSTM1 (hereafter as p62), localizes in chromosome 5 and comprises 8 exons distributed through 16 kb. The gene is conserved throughout the metazoans and its expression is ubiquitously observed 1. In response to various stresses, master gene regulators Nrf2, NF‐κB, and MiT/TFE upregulate the expression of *SQSTM1* 2, 3, 4 (Fig. 1A). Missense mutations in *SQSTM1* have been primarily associated with Paget's disease of bone, amyotrophic lateral sclerosis, and frontotemporal lobar degeneration 5. In addition, copy gains in chromosome 5q lead to clear cell renal cell carcinoma (ccRCC) at least partially due to increased expression levels of p62 6 and p62 accumulation has been observed in multiple forms of cancer (Table 1). Different p62‐positive structures such as Mallory–Denk bodies and intracellular hyaline bodies have been identified in patients suffering from steatohepatitis, alcoholic hepatitis, and hepatocellular carcinoma 7, 8.

**Table 1:** Cancer forms with described p62 accumulation

Type of cancer	References
Human hepatocellular carcinoma	42, 46, 53, 54, 90, 101, 102
Intrahepatic cholangiocarcinoma	103
Melanoma	104
Pancreatic	105
Lung	106, 107
Esophageal	108
Gastric	105, 109
Colon	105, 110
Breast	111, 112, 113, 114
Prostate	115, 116
Endometrial	117
Ovarian	118
Kidney	6
Squamous cell carcinoma of head and neck	119
Oral	120

p62, initially identified as a 62‐kDa protein 9, has multiple functional domains which include an N‐terminal Phox1 and Bem1p (PB1) domain, a zinc finger (ZZ), a tumor necrosis factor receptor‐associated factor 6 (TRAF6)‐binding (TB) motif, an LC3‐interacting region (LIR), a Keap1‐interacting region (KIR), and a ubiquitin‐associated (UBA) domain 10, 11, 12 (Fig. 1B). The protein localization is not limited to the cytoplasm, but can also be observed in the nucleus, in autophagosomes, and on lysosomes 13, 14, 15, 16. Due to its role as a receptor for selective autophagy, p62 also localizes to cargos that will be degraded during the process, such as ubiquitin‐positive protein aggregates 14, 17, 18, 19, damaged mitochondria, and invading bacteria 20. Under certain circumstances p62 can also be degraded by the proteasome 21 or endosomal‐related autophagy 22, but it is primarily degraded during selective autophagy.

p62 is known as a multifunctional signaling hub, as it participates in the activation of mTORC1 (mechanistic target of rapamycin complex 1) in nutrient sensing 15, NF‐κB activation during inflammation and apoptosis 23 and the activation of the Keap1‐Nrf2 pathway for antioxidant response 24, as well as its mentioned receptor role in selective autophagy 25. Alterations in all these pathways have been associated with cancer development 26, 27, 28, 29. Here, we describe how the interplay between p62 and the Nrf2, mTORC1, NF‐κB, and autophagy pathways plays a key cellular role both in homeostatic conditions and during cancer development.

## p62‐mediated Nrf2 activation

**Figure 2:**
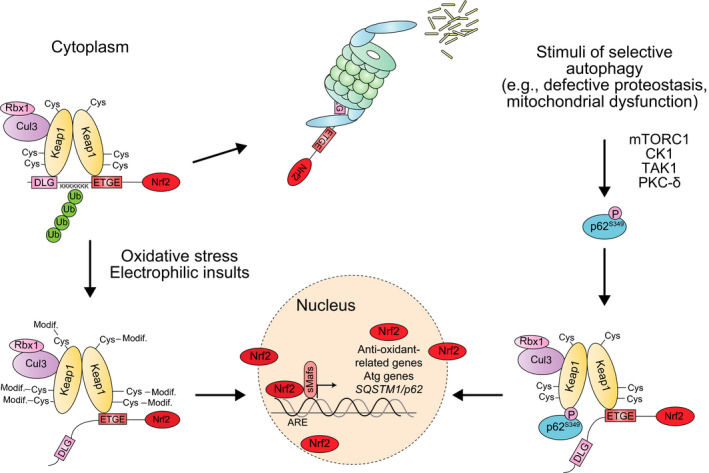
Mechanism of Nrf2 activation dependent on S349 phosphorylation of p62. Left: under basal conditions, Keap1, in collaboration with the Cul3/Rbx1 E3 ubiquitin ligase complex, promotes the proteasomal degradation of Nrf2. Upon oxidative stress, Keap1 oxidation results in the liberation of Nrf2 and its translocation to the nucleus, where it promotes the expression of a battery of target genes encoding antioxidant proteins and anti‐inflammatory enzymes. Right: phosphorylated p62 interacts with the Nrf2‐binding site of Keap1 and competitively inhibits the Keap1–Nrf2 interaction, resulting in the expression of Nrf2 target genes, which include p62. Therefore, Ser349 phosphorylated p62 causes the constitutive activation of Nrf2.

Nrf2 is a cap‘n'collar (CNC), leucine zipper (bZIP) transcription factor which controls the basal and inducible expression of over 200 genes involved in the antioxidant stress response. While Nrf2 is expressed in all cell types, its basal protein levels are usually kept low during unstressed conditions because its binding partner, Keap1, is an adaptor of Cullin3‐based ubiquitin ligases. A Keap1 homodimer recognizes a Nrf2 monomer through a two‐site ‘hinge and latch’ binding: a tight interaction with the Nrf2‐ETGE motif and a weaker one with the Nrf2‐DLGex motif, which are indispensable for sufficient ubiquitination of Nrf2. Consequently, Nrf2 undergoes proteasomal degradation, and this mechanism of regulation of Nrf2 is known as the Keap1‐Nrf2 pathway 30 (Fig. 2).

Keap1 is a cysteine‐rich protein that, upon exposure to oxidative stress or electrophilic insults, is oxidized, resulting in a conformational change in the Keap1 dimer. As a result, Nrf2 escapes from Keap1 interaction and translocates into the nucleus to induce the expression of a battery of target genes. These Nrf2 targets contain antioxidant response elements (AREs) or electrophile response elements (EpREs) in their regulatory region and encode for antioxidant and anti‐inflammatory enzymes 29 (Fig. 2).

An aberrant activation of this pathway has been observed in multiple forms of cancer 31. There are at least six alterations that can result in this activation. First, somatic mutations in the Cullin3 gene, in the coding region of Keap1 or in the DLGex and ETGE motifs of Nrf2 hamper Nrf2 recognition and degradation by the Keap1/Cul3 complex 32, 33, 34. Second, aberrant transcription of the Nrf2 gene lacking the second exon results in protein forms that cannot interact with Keap1 and constitutively localize to the nucleus 35. Third, promoter methylation of the Keap1 gene 36. Fourth, accumulation of Keap1‐binding proteins that interfere in the Keap1–Nrf2 interaction 24, 37. This mechanism will be discussed in more detail later in this section. Fifth, Keap1 oxidation by oncometabolites such as fumarate 38, 39. Sixth, transcriptional activation of the Nrf2 gene by an oncogenic KRAS mutant 40.

Besides providing increased resistance to oxidative stress, cancer cells also benefit from a metabolic reprogramming resulting from Nrf2‐persistent activation. Nrf2 regulates the expression of genes involved in glutaminolysis, purine nucleotide and glutathione synthesis, and the pentose phosphate pathway 41, 42. This establishes a feedback loop between the Keap1‐Nrf2 and the phosphatidylinositol 3‐kinase‐Akt pathways 41, 43. The latter pathway is also commonly affected in cancer 44.

As mentioned before, Nrf2 activation can also be achieved through Keap1‐binding proteins that interfere with Nrf2 degradation. p62 presents a Keap1‐interacting region (KIR) that binds to the Keap1 domain responsible for Nrf2 interaction: the Keap1 DC domain 3, 24, 45. Under basal conditions, the interaction between p62‐KIR and Keap1‐DC is too weak to compete with the interaction between Keap1‐DC and the ETGE and DLGex domains of Nrf2 46. However, p62‐KIR can undergo phosphorylation in Ser349 by multiple kinases, including mTORC1 and TGF‐β‐activated kinase (TAK1) 46, 47, 48. This modification prevents the two‐site binding between Keap1 and Nrf2, resulting in the release of Nrf2 and its translocation to the nucleus 46 (Fig. 2). Since p62 is phosphorylated at Ser349 on autophagic cargos such as ubiquitinated proteins, the Keap1‐Nrf2 pathway and selective autophagy are coupled with each other (Fig. 2 and Fig. 5C).

After its release from Keap1‐mediated degradation, Nrf2 promotes the expression of two isoforms of p62: the full‐length protein and a splicing variant lacking the KIR domain 49. We recently showed that this splicing form is still functional for autophagy but is unable to interfere in the interaction between Keap1 and Nrf2. Then, the variant form stabilizes Keap1 and suppresses the expression of Nrf2 targets (including p62) 49. Keap1 can also downregulate the p62‐Keap1‐Nrf2 pathway by promoting the ubiquitination and subsequent autophagic degradation of p62 50. Although Keap1 degradation is mainly achieved through autophagy 51, possibly in a p62‐dependent manner 3, 52, it is not clear whether the ubiquitination of p62 by Keap1 affects the autophagic degradation of Keap1 or not.

Despite these two possible mechanisms of downregulation of the p62‐Keap1‐Nrf2 pathway, the fact that p62 is a target gene of Nrf2 3 (Fig. 1A) opens the possibility for a positive feedback loop that is exploited in many forms of cancer. Table 1 shows that accumulation of p62 is a common observation in cancer cells. Phosphorylated p62 is also present in these accumulations 53, thus resulting in Nrf2 upregulation and the aforementioned metabolic reprogramming. Recent studies showed that p62 accumulation is enough to induce HCC development and confer cancer cells with enhanced proliferation capability and anticancer drug resistance due to persistent Nrf2 activation 42, 54. p62 is also a key driver of neoplastic progression in pancreas 55. Nrf2 activation derived from p62 accumulation induces MDM2, which then acts through p53‐dependent and ‐independent mechanisms to promote pancreatic ductal adenocarcinoma 55.

## mTORC1 activation by p62

**Figure 3:**
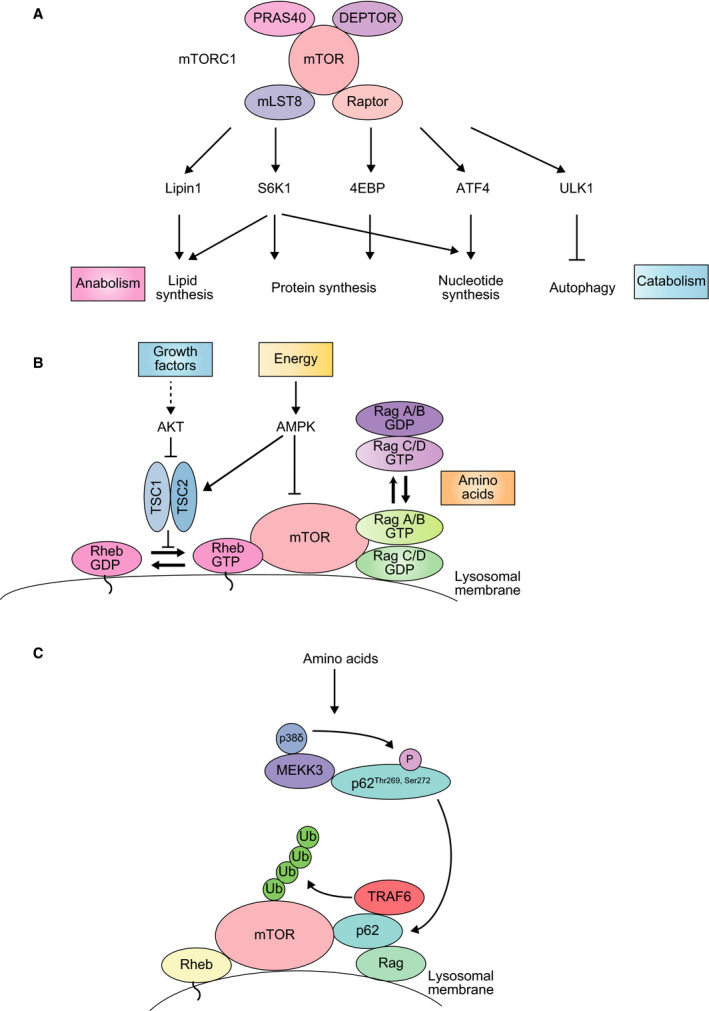
(A) The mTORC1 pathway. The mTORC1 complex is formed by the mTOR kinase, the regulator subunits Raptor and PRAS40 and the inhibitors mLST8 and DEPTOR. Upon mTORC1 stimulation, downstream effectors promote anabolism and suppress catabolism. (B) The activity of mTORC1 is inhibited by the TSC complex, which is itself inactivated in the presence of growth factors and upregulated upon energy deprivation. In the later situation, AMPK inhibits mTORC1 both directly and through TSC activation. Increased amino acid availability converts RagA/B GDP and RagC/D GTP into RagA/B GTP and RagC/D GDP respectively, assembling mTORC1 on the lysosome, where Rheb activates mTORC1. (C) mTORC1 activation by p62. Amino acid stimulation promotes the phosphorylation of p62 at Thr269 and Ser272 by MEKK3/p38delta. This results in the formation of a signaling hub over the lysosomal membrane through the interaction of p62 with Raptor, TRAF6 and Rag proteins. As a consequence, mTOR is ubiquitinated by TRAF6, and then activated by Rheb.

The mechanistic target of rapamycin is a serine/threonine protein kinase which belongs to the family of PI3K‐related kinases family. It constitutes the catalytic subunit of two distinct protein complexes, named mTOR Complex 1 (mTORC1) and 2 (mTORC2). In mTORC1, mTOR associates with Raptor, which is responsible for mTORC1 subcellular localization and substrate recruitment, and mLST8, which stabilizes the kinase activation loop of mTOR. The complex also includes two inhibitory subunits: PRAS40 and DEPTOR (Fig. 3A). Depending on the environmental conditions, cells have to fine‐tune the balance between catabolism and anabolism. In favorable conditions, mTORC1 stimulates protein, lipid, and nucleotide production to promote cell growth, concurrently blocking catabolic pathways such as autophagy. mTORC1‐dependent anabolic induction is achieved through the phosphorylation of the downstream kinase S6K1, as well as 4EBP (protein synthesis induction), Lipin1 (lipid synthesis), ATF4 (nucleotide synthesis), and HIF1α (glycolysis) (Fig. 3A). Conversely, phosphorylation of ULK1, an autophagy‐initiating kinase, by mTORC1 suppresses autophagy 26 (Fig. 3A).

Multiple signals, including growth factors, hormones, energy, and amino acid levels can regulate mTORC1 activity. In the case of growth factors, this regulation is achieved through the inhibition of mTORC1‐negative regulator TSC, resulting in mTORC1 activation by the small GTPase Rheb (Fig. 3B). Energy reduction, such as glucose deprivation, activates the metabolic regulator AMPK, which inhibits mTORC1 both directly and through TSC. On the other hand, high amino acid levels promote mTORC1 activation through Rag GTPases. These proteins, together with Rheb and the Ragulator complex, are located on the lysosomal membrane. Upon amino acid stimulation, a Rag heterodimer binds to Raptor and recruits mTORC1 to the lysosome, where it can be activated by Rheb 56, 57.

p62 was identified as an interacting partner for Raptor 15 (Fig. 1B). Further research on the topic showed that, in response to amino acid abundance, p62 is phosphorylated at Thr269 and Ser272 by its PB1‐mediated interaction with MEKK3 58. Next, p62 forms a signaling hub on the lysosomal membrane through its interaction with Raptor and the Rags proteins which recruits the ubiquitin ligase TRAF6 and mTORC1 to the surface of the organelle and, ultimately, results in K63‐linked ubiquitination of mTOR (Fig. 3C). This ubiquitination driven by p62‐TRAF6 under amino acid‐rich conditions results in mTORC1 activation, followed by coupling nutrient availability to anabolism and cell growth 15, 58, 59.

The enhanced proliferative rate of cancer cells is highly benefited by mTORC1 upregulation 26. Thus, upstream regulators of mTOR, such as PTEN and Akt, are frequently mutated in human tumors, and MTOR mutations are also found in some cancer subtypes 26, 60. p62 is accumulated in multiple forms of cancer (Table 1), and it is likely exerting its carcinogenic role through enhanced mTORC1 activation. In agreement with this, HCC developed by TSC depletion (which results in constitutive mTORC1 activation) is reverted by concurrent deletion of p62 54. But, while tumor cells benefit from increased levels of p62, they also take profit from p62 downregulation in the surrounding stromal cells, a phenomenon commonly observed in prostate and liver 11. In prostate cancer, loss of p62 in the stromal fibroblasts impairs mTORC1 activation, which results in a reduced metabolic detoxification capacity and the secretion of the prosurvival inflammatory cytokine IL‐6, which sustains the growth and invasiveness of the neighboring prostate cancer cells 61. In addition, this loss of p62 in stromal cells also promotes the accumulation of ATF4 (a downstream effector of mTORC1 that undergoes proteasomal degradation in a p62‐dependent manner), starting a transcriptional program that delivers asparagine to the cancer cells, allowing them to survive in conditions of glutamine deprivation 62. This asparagine also ensures mTORC1 activation in the tumor, sustaining the proliferative anabolic signaling in cancer cells 63, 64.

## Regulation of NF‐κB signaling through p62

**Figure 4:**
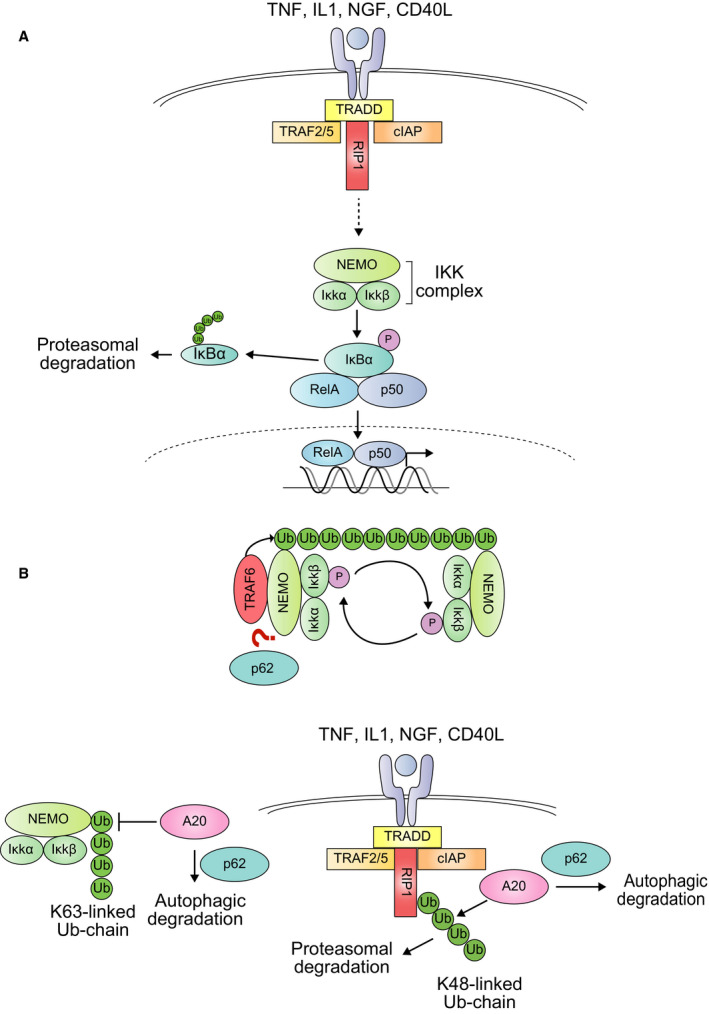
(A) The NF‐κB pathway. Multiple signals, such as proinflammatory cytokines or growth factors, can induce NF‐κB activation. Following the ligand‐receptor binding, the activation of the IKK complex releases NF‐κB (RelA–p50 complex) from its inhibitor IκB. This is achieved through the phosphorylation and subsequent proteasomal degradation of the inhibitor. Then, NF‐κB translocates to the nucleus and induces the expression of target genes, including p62. (B) Two mechanisms of p62‐mediated NF‐κB activation. First, p62 seems to be required for NEMO ubiquitination by TRAF6. This ubiquitination is recognized by the NEMO subunits of other IKK complexes, bringing them into the close proximity required for their cross‐phosphorylation and activation. Second, p62 promotes the autophagic degradation of the ubiquitin‐editing enzyme A20, which otherwise inhibits NF‐κB activation by promoting RIP1 degradation and reversing NEMO ubiquitination.

The NF‐κB is a key family of regulators of inflammation and the immune response that is composed by five members combined into homo‐ or heterodimers. There are two main kinds of NF‐κB signaling pathways: the classical, in which proinflammatory cytokines promote a rapid and transient induction, and the alternative, which depends on *de novo* synthesis of the NF‐κB‐inducing kinase NIK, and then requires a longer activation time. In both cases, the activation is achieved through the degradation of specific inhibitors, the IκB proteins, once they are phosphorylated by an IKK complex 65 (Fig. 4A).

Classically, the IKK complex is composed by the catalytic subunits IKKα and IKKβ together with the regulatory subunit NEMO (or IKKγ). The activation of the complex requires the trans‐autophosphorylation between two adjacent IKK catalytic subunit dimers 27 (Fig. 4B). These dimers are brought together by the nondegradative ubiquitination of their NEMO subunits, which can bind to the ubiquitin chain of the other regulatory subunit 66. This nondegradative ubiquitination, mainly composed of K63‐ and M1‐linked chains, is catalyzed by E3 ubiquitin ligases such as TRAF6 and LUBAC, respectively. In the case of TRAF6, p62 seems to participate in the K63‐linked ubiquitination of NEMO 67 (Fig. 4B). An additional role of p62 in NF‐κB activation is to promote the degradation of A20, a ubiquitin‐editing enzyme that prevents excessive NF‐κB activation by counteracting K63‐linked ubiquitination of NEMO and promoting K48‐linked ubiquitination of RIP1, followed by its degradation by the 26S proteasome 68, 69. At the same time, p62 is upregulated by NF‐κB, creating a positive feedback loop 2 (Fig. 1A).

The NF‐κB pathway participates in every step of tumor formation. First, it promotes the production of reactive oxygen species (ROS) that induce DNA damage and oncogenic mutations. Second, it enhances the proliferation of these cells, while preventing p53‐induced apoptosis. Finally, inflammation and NF‐κB support malignant progression by stimulating the expression of genes that enhance neoangiogenesis and the invasive behavior of the cancer cells 70, 71. Pancreatic ductal adenocarcinoma (PDAC) and lung cancer are two examples in which KRAS mutation results in a feedforward loop between NF‐κB and p62 that promotes tumorigenesis through the aforementioned mechanisms 2, 72.

During cancer development, macrophages are recruited to the tumor microenvironment to protect the organism by engulfing the damaged cells. Induction of p62 through NF‐κB in macrophages seems to be required to limit inflammasome‐mediated IL‐1β release. Then, p62 protects macrophages from excessive NF‐κB activation, and genetic ablation of p62 in these cells results in increased tissue damage and IL‐1β‐dependent inflammation, which is likely to promote tumorigenesis 73, 74. Likewise, as mentioned in a previous section, cancer‐associated fibroblasts develop an aberrant phenotype due to their reduced levels of p62. These fibroblasts maintain a prosurvival transcriptional program that increases the secretion of proinflammatory cytokines. These paracrine signals stimulate tumorigenesis and cancer progression at least partly in an NF‐κB‐dependent manner 61, 75, 76.

## Autophagy and p62

**Figure 5:**
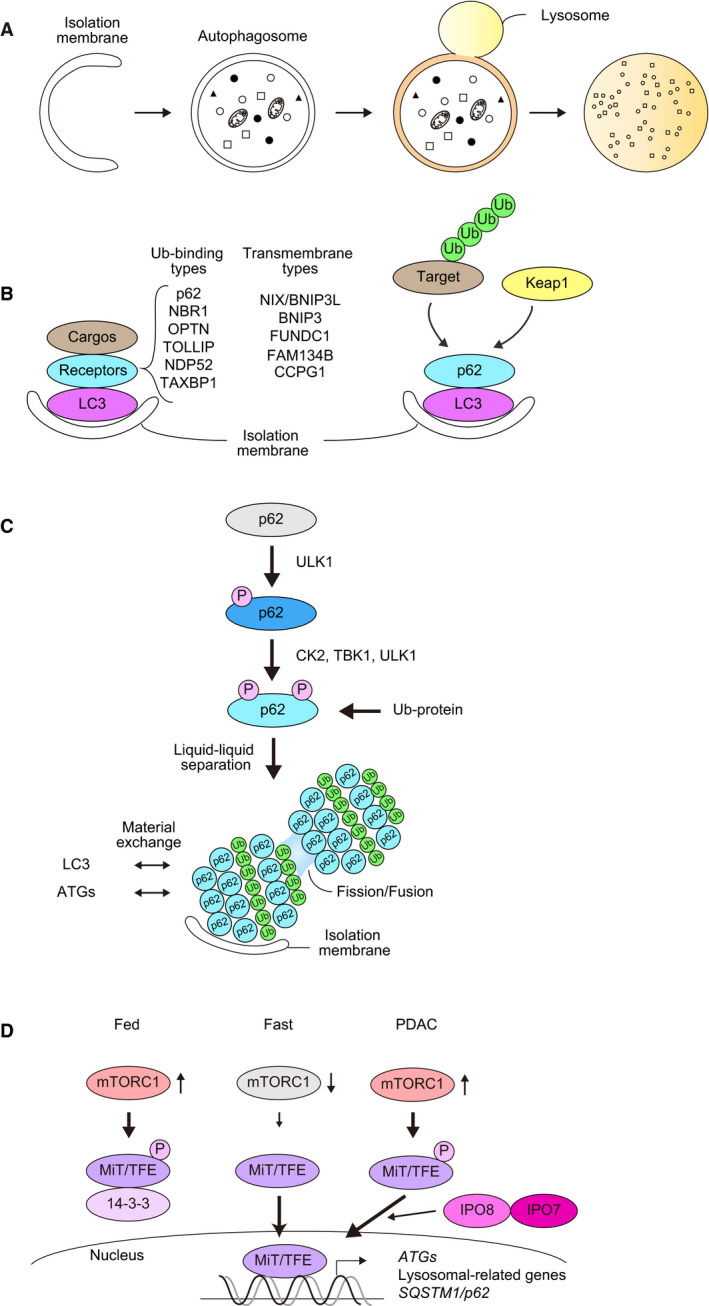
(A) Macroautophagy is accompanied by dynamic membrane biogenesis and autophagosome formation. The autophagosome sequesters a portion of the cytoplasm and fuses with the lysosome, where its contents are degraded. (B) Receptor‐mediated selective autophagy. Receptor proteins are divided into two groups: ubiquitin‐binding type and transmembrane type. Both have the ability to bind to Atg8 family proteins (e.g., LC3s and GABARAPs). p62 is a representative Ub‐binding type receptor, and it also mediates autophagic degradation of certain proteins (e.g., Keap1) through their direct interaction without prior ubiquitination. (C) p62‐mediated selective autophagy. When selective autophagic cargos such as misfolded proteins appear in the cytoplasm, they are ubiquitinated. Concomitantly, Ser407, located at the UBA domain of p62, is initially phosphorylated by ULK1 kinase. This phosphorylation destabilizes the UBA dimer interface and subsequently casein kinase 2 (CK2), TANK‐binding kinase 1 (TBK1), or ULK1 phosphorylate Ser403 of the UBA domain, which increases the binding affinity of p62 for the ubiquitin chain. The p62 forming a complex with ubiquitin chains has liquid‐like properties and forms droplets in which LC3 and other Atg proteins assemble to form the isolation membrane. Keap1, a binding protein for p62 may also shuttle from cytoplasm to the droplets. (D) Regulation of the nuclear translocation of the MiT‐TFE family by mTORC1‐mediated phosphorylation. Under nutrient‐rich conditions, the MiT‐TFE family is phosphorylated by mTORC1 and subsequently trapped by 14‐3‐3 protein. As a result, the MiT‐TFE family is kept in the cytoplasm. Once mTORC1 is inactivated by metabolic stresses such as nutrient deprivation, the MiT‐TFE family translocate into the nucleus to induce lysosomal biogenesis, Atg genes and *p62*/*SQSTM1*. In PDAC, increased level of Importin8 and 7 bypasses the regulation of mTORC1.

Autophagy is defined as an intracellular lysosomal degradation pathway. Among its different subtypes, macroautophagy (thereafter referred to as autophagy) is the most characterized form of autophagy and is defined by the formation of a double membrane structure called the autophagosome that will later fuse with the lysosome 77 (Fig. 5A). There are two modes of autophagy: nonselective and selective autophagy, in which the autophagosome either randomly sequesters cytoplasmic components or targets specific cargos, respectively. When specific autophagic cargos such as damaged or excess organelles appear in the cytoplasm, they are tagged with ubiquitin, leading to the assembly of receptor proteins that bind to the ubiquitin chain (Ub‐binding type receptors, Fig. 5B). Alternatively, transmembrane type of receptor proteins directly localize on cargos (transmembrane‐type receptors, Fig. 5B). In the latter case, molecular markers like ubiquitin are not needed. Most receptor proteins have LC3‐interacting regions, called LIRs, to interact with ATG8 family proteins 25, 78. Thus, cargo labeling and the transfer of receptor proteins to cargos mainly regulate selective autophagy (Fig. 5B). Several receptor proteins are delivered to the lumen of autophagosomes together with ATG8 family proteins and degraded in lysosomes, being themselves targets of selective autophagy.

p62 is the best characterized receptor protein for selective autophagy of ubiquitinated cargos 10 (Fig. 5B). When its activity as an autophagy receptor is not required, p62 is held inactive through homodimerization of its UBA domain, which prevents it from interacting with ubiquitin 79, 80. Phosphorylation events drive the liberation of the UBA domain from dimeric inactivation; in particular, phosphorylation of serine 407 of p62 by ULK1 has been shown to facilitate the transition from dimer to monomer 81. This modification is followed by phosphorylation of p62 on serine 403, either by ULK1, casein kinase 2 (CK2) or TANK‐binding kinase 1 (TBK1), which enhances its binding to ubiquitin chains 82, 83, 84 (Fig. 5C). Once p62 binds to ubiquitin chains, it acquires liquid‐like properties 85, 86. Such phase‐separated droplets allow the exchange of their components, including ubiquitin and LC3, with the surrounding environment. Consequently, the droplets can also function as nodes from which signaling cascades can be activated in the context of selective autophagy (Fig. 5C). The droplet formation is rendered more efficient by the presence of NBR1, another autophagic receptor that cooperates with p62 in cells 86. Considering the analogy of APE1 transport into vacuole through selective autophagy in yeast 87 and the cases of parkin‐mediated mitophagy 88 and CCPG1‐mediated ER‐phagy 89, upstream autophagy factors such as the ULK1‐kinase complex as well as p62 binders such as Keap1 might also be translocated onto the droplets. In agreement with this hypothesis, Keap1, a p62‐interacting protein involved in Nrf2 signaling 50, has been found to localize to these droplets 3, 24, 52. Once the pathway is activated, p62‐positive structures together with the recruited cargo are degraded by autophagy.

Autophagy impairment caused by the loss of *Atg5* or *Atg7* in mouse liver is usually accompanied by accumulation of degenerated protein aggregates, damaged mitochondria, peroxisomes, and lipid droplets, as well as persistent activation of Nrf2 due to the sequestration of Keap1 into p62‐positive structures (refer to the Nrf2 section), leading to benign adenoma 90, 91. Mice haploinsufficient for the autophagy gene Beclin 1 are also cancer prone; they develop spontaneous tumors, including HCC, and are highly susceptible to HCC upon infection with HBV 92, 93. The primary cause of such tumorigenesis is probably ROS derived from degenerated mitochondria and/or peroxisomes, leading to genomic instability and ultimately to spontaneous oncogenesis. Simultaneous loss of *p62* or *Nrf2* in mice with livers deficient in *Atg7* or *Atg5* suppresses tumor development 91, 94, implying that the p62‐mediated activation of Nrf2 contributes to tumor growth in autophagy‐deficient mouse livers.

During the first steps of cancer initiation, autophagy is thought to be tumor suppressive through its cytoprotective function 95. However, once the tumor has originated, autophagy is induced and protects cancer cells from metabolic stress, inflammation, and genotoxic and therapy‐induced stresses. Indeed, loss of autophagy suppresses tumor development in various mouse models including nonsmall‐cell lung cancer, pancreatic ductal adenocarcinoma, melanoma, and prostatic carcinoma 95. While dephosphorylation of ULK1 through inactivation of mTORC1 is needed for the induction of autophagy in response to nutrient deprivation, transcriptional activation of genes involved in lysosomal biogenesis and *Atg* genes is also indispensable for prolonged stimulation of autophagy, which is usually observed in tumor cells. The expression of such genes is mainly regulated by the basic helix‐loop‐helix transcription factors of the MiT‐TFE family (MITF, TFE, and TFEB) 4. In nutrient‐rich conditions, the MiT‐TFE family is phosphorylated by mTORC1 and retained in the cytoplasm 96 (Fig. 5D). When mTORC1 is inactivated under nutrient‐poor conditions, the MiT‐TFE family is dephosphorylated and then translocated into the nucleus in order to trans‐activate the genes harboring a coordinated lysosomal expression regulation (CLEAR) element in their promoter regions (Fig. 5D). In PDAC, the MiT‐TFE family is constantly localized in the nucleus even in mTORC1‐activating conditions. This has been attributed to enhanced transport of the MiT‐TFE family to the nucleus due to increased level of the nuclear transporters Importin 7 and 8 97 (Fig. 5D). In addition, phosphorylation of ULK1 by mTORC1, which leads to suppression of autophagy, is inhibited due to hyperactivation of the responsible phosphatase, B55α‐PP2A, in PDAC 98. Since *p62/SQSTM1* is also a target gene for the TiF‐TFE family as well as Nrf2 and NF‐κB, both of which are usually activated in tumor cells, the p62 protein might be increased in certain types of cancers even under autophagy‐inducing conditions.

## Concluding remarks

Evaluation of biological complexity has been an elusive question for decades. It is now increasingly clear that the connectivity of regulatory networks rather than the sheer number of genes or the genomic size is what determines how complex an organism is 99. In the case of p62 it is not the many pathways in which it is involved, but how they are entangled what truly represents the beauty of molecular complexity. Focusing on the processes described here, p62 upregulation of mTORC1 upon amino acid abundance provokes an inhibition of autophagy, which results in increased levels of p62 100. At the same time, mTORC1 is responsible for p62 activation by phosphorylation during stress conditions, which directly results in Nrf2 activation 46. p62 and TRAF6 seem to collaborate in the activation of mTORC1 59. p62 and TRAF6 also cooperate in the activation of the NF‐κB pathway. In addition, both NF‐κB and Nrf2 upregulate the expression of p62, and can enter as a consequence in a feedforward loop 2, 3. Whether the four major pathways described here are the only ones subjected to regulation by p62 or not still requires further research. What is increasingly clear is that, just by functioning as an adaptor of protein signaling and controlling the autophagic degradation of some of its binding partners, p62 masters a wide biological network affecting multiple and sometimes unrelated processes.

Cancer cells benefit from increased levels of p62 (that may be caused by autophagy impairment), as it provides them with an improved antioxidant and detoxifying response (Nrf2 activation) and proangiogenic and prosurvival signals (NF‐κB activation), both of which promote tumor progression. Accumulation of p62 may also cause a switch toward anabolism (mTORC1 activation), although this connection needs to be further explored. At the same time, loss of p62 in the surrounding stromal cells, and thereby downregulation of these pathways, results in the secretion of metabolites and paracrine signals that further help cancer thriving. Curiously, although the mechanisms discussed in this review support that both up‐ and downregulation of p62 can promote cancer, p62 mutations are primarily linked to skeletal and neurodegenerative diseases, with only one report linking copy gains of p62 with kidney cancer 6. Still, being at the crossroads of so many cellular pathways, any temptation of employing p62 as a therapeutic target has to be counterbalanced with the myriad of possible side effects in nonconsidered scenarios.

## Author contributions

MK and TS designed the review contents. TS and PS‐M created the Figures. All authors wrote the manuscript.
